# B Cell Signatures Distinguish Cutaneous Lupus Erythematosus Subtypes and the Presence of Systemic Disease Activity

**DOI:** 10.3389/fimmu.2021.775353

**Published:** 2021-11-19

**Authors:** Lisa Abernathy-Close, Stephanie Lazar, Jasmine Stannard, Lam C. Tsoi, Sean Eddy, Syed M. Rizvi, Christine M. Yee, Emily M. Myers, Rajaie Namas, Lori Lowe, Tamra J. Reed, Fei Wen, Johann E. Gudjonsson, J. Michelle Kahlenberg, Celine C. Berthier

**Affiliations:** ^1^ Division of Rheumatology, Department of Internal Medicine, University of Michigan, Ann Arbor, MI, United States; ^2^ Department of Pediatrics, University of Michigan, Ann Arbor, MI, United States; ^3^ Department of Dermatology, University of Michigan, Ann Arbor, MI, United States; ^4^ Department of Computational Medicine & Bioinformatics, University of Michigan, Ann Arbor, MI, United States; ^5^ Department of Biostatistics, University of Michigan, Ann Arbor, MI, United States; ^6^ Division of Nephrology, Department of Internal Medicine, University of Michigan, Ann Arbor, MI, United States; ^7^ Department of Chemical Engineering, University of Michigan, Ann Arbor, MI, United States; ^8^ Lifebridge Health, Baltimore, MD, United States; ^9^ Division of Rheumatology, Department of Internal Medicine, Cleveland Clinic Abu Dhabi, Abu Dhabi, United Arab Emirates; ^10^ Department of Pathology, University of Michigan, Ann Arbor, MI, United States

**Keywords:** lupus, discoid, B cells, transcriptomic, cutaneous lupus, autoantibodies

## Abstract

Cutaneous lupus erythematosus (CLE) is a chronic inflammatory skin disease characterized by a diverse cadre of clinical presentations. CLE commonly occurs in patients with systemic lupus erythematosus (SLE), and CLE can also develop in the absence of systemic disease. Although CLE is a complex and heterogeneous disease, several studies have identified common signaling pathways, including those of type I interferons (IFNs), that play a key role in driving cutaneous inflammation across all CLE subsets. However, discriminating factors that drive different phenotypes of skin lesions remain to be determined. Thus, we sought to understand the skin-associated cellular and transcriptional differences in CLE subsets and how the different types of cutaneous inflammation relate to the presence of systemic lupus disease. In this study, we utilized two distinct cohorts comprising a total of 150 CLE lesional biopsies to compare discoid lupus erythematosus (DLE), subacute cutaneous lupus erythematosus (SCLE), and acute cutaneous lupus erythematosus (ACLE) in patients with and without associated SLE. Using an unbiased approach, we demonstrated a CLE subtype-dependent gradient of B cell enrichment in the skin, with DLE lesions harboring a more dominant skin B cell transcriptional signature and enrichment of B cells on immunostaining compared to ACLE and SCLE. Additionally, we observed a significant increase in B cell signatures in the lesional skin from patients with isolated CLE compared with similar lesions from patients with systemic lupus. This trend was driven primarily by differences in the DLE subgroup. Our work thus shows that skin-associated B cell responses distinguish CLE subtypes in patients with and without associated SLE, suggesting that B cell function in skin may be an important link between cutaneous lupus and systemic disease activity.

## Introduction

Systemic lupus erythematosus (SLE) is complex, chronic, autoimmune disease characterized by hyperreactive B cells and the production of pathogenic autoantibodies (1). SLE involves multiple organ systems, including the skin, where the distinct type of inflammation is termed cutaneous lupus erythematosus (CLE). CLE can occur in isolation or as a skin manifestation associated with underlying systemic lupus erythematosus (SLE) (2). CLE is relatively understudied compared to SLE, which contributes to a lack of understanding of disease heterogeneity in CLE pathogenesis. CLE is a rubric which encompasses clinically and histologically distinct subtypes of CLE: acute cutaneous lupus erythematosus (ACLE), subacute cutaneous lupus erythematosus (SCLE), or chronic lupus erythematosus (CCLE), with discoid lupus erythematosus (DLE) being the most common subtype (3–5). While there are consistently observed cellular and molecular features in patients with CLE and/or SLE, such as a type I interferon (IFN) gene signature in the blood and skin (6–10) and peripheral B cell dysfunction (11, 12), the shared and unique molecular and cellular features of ACLE, SCLE, and CCLE remain poorly understood. Indeed, basic transcriptional comparisons have not identified robust distinguishing molecular signatures between subtypes (13, 14). Further, DLE is more likely to occur without underlying SLE compared to ACLE or SCLE (2, 15), yet it is not clear if the presence or absence of systemic disease is related to the differences observed in cutaneous manifestations of lupus (16).

In this study, we sought to investigate cellular and transcriptional differences in lesional skin biopsies across CLE subtypes, including DLE, ACLE, and SCLE, and explore how cutaneous lesional immunophenotypes relates to systemic lupus disease. Using novel analyses, we found that DLE lesions harbor a unique immunoglobulin signature and an enrichment of skin B cells compared to ACLE or SCLE lesions. Intriguingly, this B cell signature was highest in patients with CLE without concomitant SLE, including within the entire cohort of DLE patients (2). Our results demonstrate that a B cell gene signature in the skin distinguishes DLE from ACLE and SCLE and that increased B cells in the skin of DLE patients is indicative of a lower rate of accompanying systemic disease. These data suggest that B cell transcriptional programs are more activated in DLE lesions relative to ACLE or SCLE lesions and may play a role in immunopathogenesis divergence across CLE subtypes. This work supports future exploration of utilizing a skin B cell score as a clinical marker of SLE risk, especially in DLE patients.

## Materials and Methods

### Study Design

We applied a tissue transcriptome-driven sequential analysis strategy. Gene expression profiles from 90 cases of CLE that include DLE (n=47) and SCLE (n=43), as well as 13 healthy control skin biopsies were used as a discovery cohort (13). Subsequent profiles of 60 skin biopsies that include DLE (n=20), SCLE (n=20) and ACLE (n=20) served as a validation cohort along with 4 additional healthy control skin biopsies. The details of the discovery cohort and sample collection protocol are previously described (13). In brief, skin biopsies were identified *via* a SNOMED search of the University of Michigan Pathology Database using the search terms “lupus” and “cutaneous lupus”. Patients who met both clinical and histologic criteria for DLE or SCLE or ACLE were included in the study. Patients with drug-induced CLE were excluded from this study. The average time from diagnosis to skin biopsy ranged from two to four years for patients in the discovery cohort. Gender-, age- and race-matched healthy controls were identified and utilized for studies that compared CLE to normal healthy control skin (n=13 in the discovery cohort, n=4 in the validation cohort). Clinical data information can be found in **Supplementary Table 1**. Systemic disease was defined by ACR criteria ≥4 (17).

### Gene Expression Analysis

For both discovery and validation cohorts, transcriptome analysis was performed on skin biopsies using Affymetrix ST2.1 GeneChips as previously published (13). Data processing details for the discovery cohort can be found in Berthier et al. (13). In brief, normalized expression data were log2-transformed and batch-corrected. FDR was applied to account for multiple testing. The CEL-files and processed data are available at Gene Expression Omnibus (https://www.ncbi.nlm.nih.gov/geo/) under the reference number GSE81071 (discovery cohort) and GSE184989 (validation cohort).

For the validation cohort, samples were processed and normalized using the RMA approach (18), and average expression was taken if more than one probesets mapping to a gene. We then estimated and controlled the latent confounding variables (19) for the limma-based differential expression analysis (20, 21).

### IFN Score Calculation

For both discovery and validation cohorts, IFN score was calculated from the gene expression data as previously described (13).

### Weighted Gene Co-Expression Network Analysis

Weighted gene co-expression network analysis (WGCNA) (22) was performed on the 20,410 genes of the discovery cohort. Briefly, WGCNA was used to aggregate genes into co-expression modules representing gene expression patterns across all patient samples. Co-expression modules were named and represented by a unique color. A module eigenene value (the first principal component of each module gene set) was generated in each sample used as representation of the module. Each module eigengene value was associated with available clinical variables: Cutaneous Lupus Erythematosus Disease Area and Severity Index (CLASI), Systemic Lupus Erythematosus Disease Activity Index (SLEDAI), systemic versus non-systemic disease status, DLE versus SCLE status, and IFN score.

### Heatmap Generation, Cell Type Enrichment and Literature-Based Pathway Analyses

Heatmaps of gene expression datasets were generated using the Morpheus software (https://software.broadinstitute.org/morpheus). Cell type enrichment analysis was performed as previously reported (13) on the normalized datasets of 20,410 genes (discovery cohort) and of 29,405 genes (validation cohort) using the xCell webtool (http://xcell.ucsf.edu/) (23). Canonical pathways were identified using Ingenuity Pathway Analysis software (IPA) (www.ingenuity.com).

### Tissue CyTOF

Formalin-fixed, paraffin-embedded (FFPE) skin biopsy tissue sections from lesional skin of patients with ACLE, SCLE, or DLE were analyzed using the Hyperion imaging CyTOF system (Fluidigm) as previously described (24) with modifications of the antibody panel. Specifically, metal-tagged antibodies including pan-keratin (C11, Biolegend), BDCA2 (Polyclonal, R&D Systems), CD56 (123C3, ThermoFisher Scientific), HLA-DR (LN3, Biolegend), CD11c (EP1347Y, Abcam), and CD4 (EPR6855, Fluidigm) were added in this study.

### Immunohistochemistry

CLE skin biopsies from lesions of patients with ACLE, SCLE, or DLE as well as healthy controls were collected and fixed in formalin. Formalin-fixed, paraffin-embedded skin biopsy sections were assayed by chromogenic immunostaining for pan-leukocytes (anti-CD45, HI30, eBioscience), B cells (anti-CD20, L26, Abcam) and memory B cells (anti-CD27, polyclonal, R&D Systems). Antigen retrieval was achieved by heating sections in sodium citrate buffer (pH 6.0) prior to antibody incubation. A minimum of 3 patients per disease status group were assayed and representative images are shown.

### Statistical Analyses

Statistical analysis of clinical data and gene score comparisons were generated using an unpaired parametric t-test with GraphPad Prism software version 8.0.0; p-values<0.05 were considered statistically significant and reported in all Figures. All comparisons across all groups were performed; for clarity, only the most relevant were reported if statistically significant.

## Results

### DLE Lesions Harbor a Unique Skin Immunoglobulin Gene Signature Compared to SCLE Lesions

**Figure 1:**
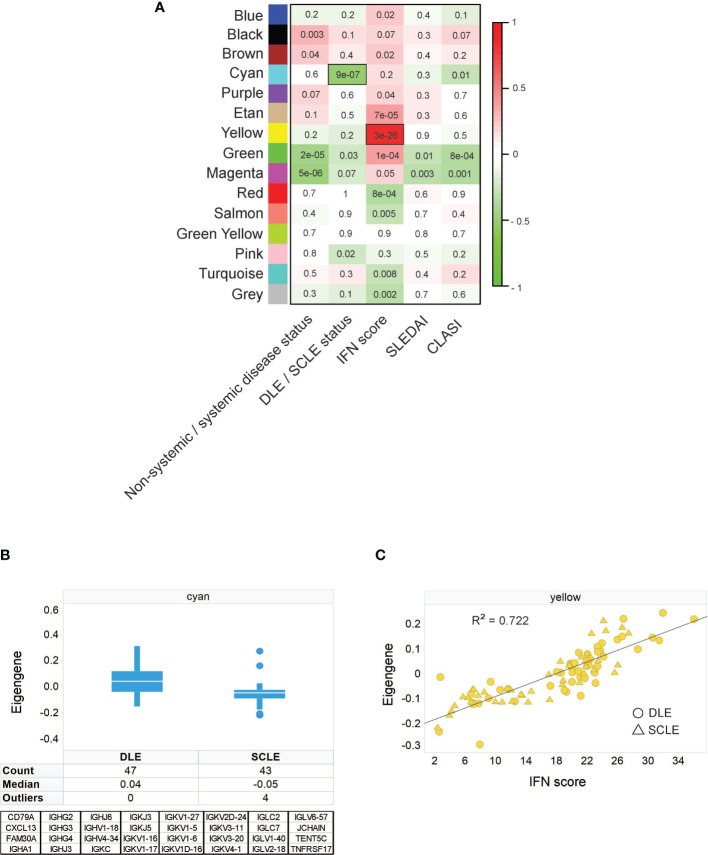
Weighted gene correlation network analysis identifies an immunoglobulin signature associated with DLE skin lesion status, independent of IFN score. **(A)** Module-trait relationship heatmap. Each module eigengene was correlated with the indicated clinical parameter. For categorical parameters, non-systemic/systemic disease and DLE/SCLE, numerical values were assigned to each categorical group. The scale bar on the right represents the correlation coefficient with green for negative correlation and red for positive correlation, p-values for each correlation are presented on the heatmap. The yellow module was the module with the strongest positive correlation with IFN score and the cyan module was the module that had the strongest negative correlation with the DLE versus SCLE lesion status. **(B)** The cyan module eigengene from the SCLE and DLE lesions encompasses a 32-gene, primarily immunoglobulin signature. **(C)** The yellow module eigengene was significantly correlated with IFN score (r = 0.85, p = 3E-28). The data in each panel represent 47 DLE patients and 43 SCLE patients.

To identify unique features amongst CLE subtypes, weighted gene correlation network analysis (WGCNA) was performed on an initial discovery skin cohort to identify modules of genes correlating with available patient clinical variables: CLASI, SLEDAI, systemic versus non-systemic disease status, DLE versus SCLE status, and IFN score. The resulting modules were categorized by color and correlations of each module eigengene with clinical variables depicted in the module-trait relationship heatmap **(** **Figure 1A**). This analysis identified that the cyan module was one of the modules with the strongest correlation with clinical variables and was significantly higher in DLE compared to SCLE status (**Figure 1B**). This cyan module was775353 composed of 32 genes, 26 of which were immunoglobulin genes (**Figure 1B**). This result was confirmed in a separate validation cohort that also included patients with ACLE (**Supplementary Figure 1A**). Further probing by Ingenuity pathway analysis also revealed significant enrichment for several B cell-related pathways (**Supplementary Table 2**) including B cell receptor signaling (p=6.31x10^-33^), B cell signaling pathway (p=1.58x10^-31^), B cell activating factor signaling (p=4.79x10^-02^), and B cell development (p=4.79x10^-02^). The yellow module from the WGCNA analysis, represented by 746 genes (**Supplementary Table 3** **),** was significantly correlated with the increased IFN score in both the CLE discovery cohort (**Figure 1C**, r^2 ^= 0.722, p=3x10^-28^) and validation cohort (**Supplementary Figure 1B**, r^2^ = 0.832, p<0.0001). These data demonstrate that a stronger immunoglobulin gene signature was observed in lesional skin from patients with DLE when compared to lesions from those with SCLE, and that a high skin IFN score was correlated with active CLE lesions, regardless of cutaneous disease subtype.

### DLE Lesions Are Associated With a Higher Skin B Cell Signature Compared to ACLE or SCLE

To explore whether the skewed cutaneous immunoglobulin gene signature detected in DLE lesions coincided with an increase in skin B cell subsets compared to other CLE subtypes, we utilized the xCell algorithm which performs cell type enrichment analysis from tissue gene expression profiles (23). The heterogeneous cellular landscape of tissue expression profiles can be evaluated with the xCell enrichment scores generated for each cell type. We performed this analysis on both the discovery and the validation cohorts which included normalized gene expression from the skin of healthy controls or lesions from patients with DLE, SCLE, or ACLE. (**Supplementary Figure 2** and **Supplementary Table 4**).

**Figure 2:**
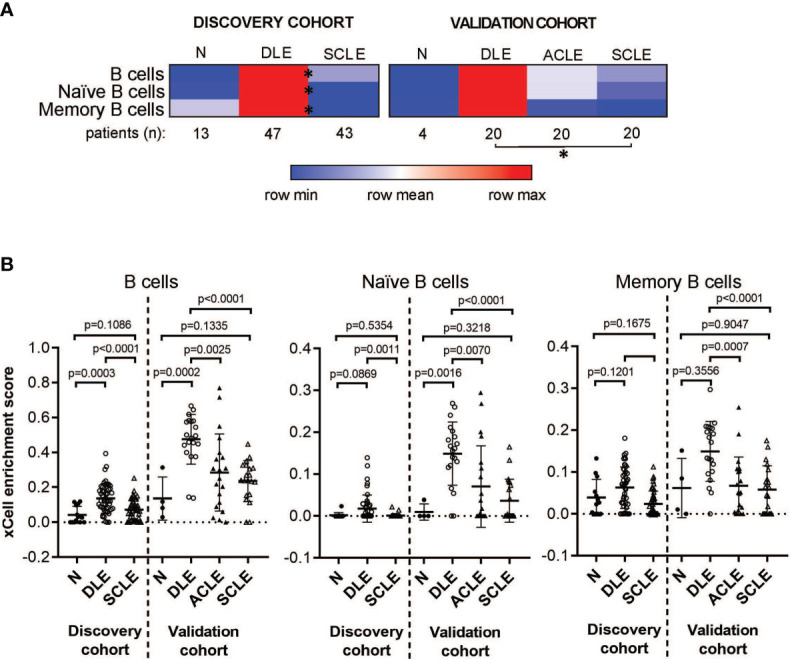
Cell type enrichment analysis using xCell tool reveals a B cell signature higher in DLE lesional skin compared to SCLE and ACLE lesions. **(A)** Heatmap of the B cell subtypes representing average xCell score for each skin lesion type compared to normal healthy controls (N). *p-value < 0.05 in SCLE versus DLE. **(B)** xCell enrichment score for B cells, naïve B cells and memory B cells in lesional skin from patients with DLE, ACLE, SCLE as well as normal healthy controls (N) in both the discovery and the validation cohort. Comparisons were made *via* unpaired Students’ t-test.

The cell type enrichment analysis showed enrichment for B cell subsets in patients with DLE compared to SCLE or ACLE skin lesions (**Figure 2A**). Specifically, cell type enrichment analysis from both the discovery and validation cohorts revealed significantly higher gene expression signatures for B cells (p=0.0001 and p<0.0001, respectively), naïve B cells (p=0.0011 and p<0.0001, respectively) and memory B cells (p<0.0001 and p=0.0001, respectively) in skin lesions from patients with DLE compared to SCLE (**Figure 2B**). Furthermore, B cell enrichment scores of B cells, naïve B cells, and memory B cells were all significantly lower in ACLE lesions compared to DLE (**Figure 2B**, p=0.0025, p=0.0007, p=0.0070, respectively). No significant difference in the enrichment of B cell subsets between lesions from patients with SCLE and ACLE was detected by cell type enrichment analysis (**Figure 2B**). These results show that when CLE is examined according to cutaneous disease subtype, a significant enrichment in overall and subsets of skin B cell gene expression programs is detected in DLE lesions compared to SCLE or ACLE lesions.

### Skin B Cell Enrichment in CLE Is Associated With Non-Systemic Lupus and Discoid Lesions

**Figure 3:**
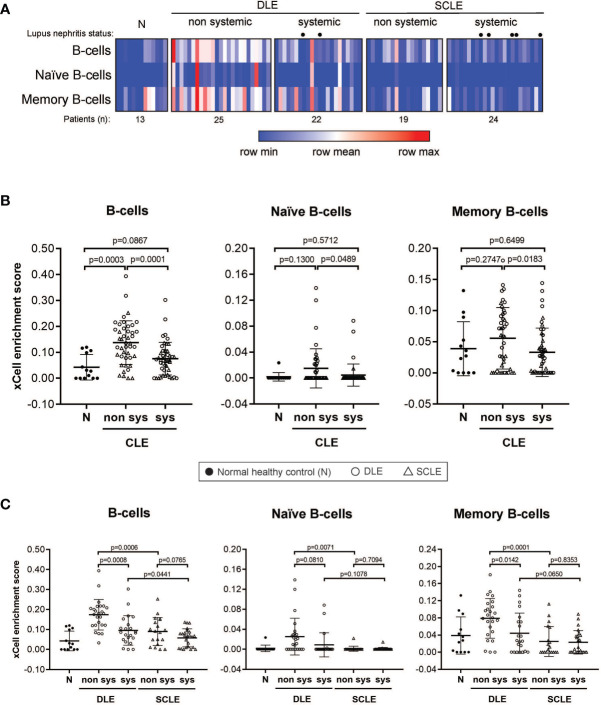
B cell subset enrichment score in lesional skin from CLE patients with and without systemic lupus. **(A)** Heatmap of the B cell subtypes representing the xCell enrichment score for each patient from the discovery cohort. **(B)** xCell enrichment score for B cell subtypes in normal healthy controls (N) (n = 13) and all CLE patients with and without systemic lupus (n = 46 and n = 44, respectively). **(C)** xCell enrichment score for B cell subtypes in DLE patients with and without systemic lupus (n = 22 and n = 25, respectively) and SCLE patients with and without systemic lupus (n = 24 and n = 19, respectively). Comparisons were made *via* unpaired Students’ t-test.

We then sought to determine if the B cell signature detected in CLE lesions was associated with systemic disease status, including within a particular CLE subtype. For this, we first grouped all CLE lesions together and analyzed them based on whether the patient had systemic SLE or CLE without systemic disease. Intriguingly, we identified a significant difference in lesional B cell subsets vs. healthy control only in patients with CLE without associated SLE (p=0.0003). No increase in B cell subsets were noted when lesions from SLE patients were compared with healthy controls (**Figure 3A**). This trend was less when B cells were subsetted into naïve and memory, but in all instances, CLE lesions taken from patients without SLE exhibited significantly more B cell-associated gene expression. (**Figure 3A**). Because we observed differences in the lesional skin B cell enrichment score in patients with CLE based on SLE status, we further explored the impact of CLE subtype on this finding. There were significantly higher total B cell enrichment scores in lesions from patients with isolated DLE compared to DLE with underlying SLE (p=0.0008). A similar trend was seen for SCLE but this did not reach significance (p=0.077) (**Figure 3B**). This B cell enrichment was also specific to non-systemic DLE versus non-systemic SCLE when total B cells (p=0.0001), naïve B cells (p=0.0489), and memory B cells (p=0.0183) were compared (**Figure 3**). Thus, lack of associated SLE is associated with increased B cell signatures in CLE lesions, and this holds true even within the DLE subtype when patients with and without associated SLE are compared.

### Peripheral Autoantibody Status Is Related to the Degree of B Cell Enrichment in Cutaneous Lupus Lesions

**Figure 4:**
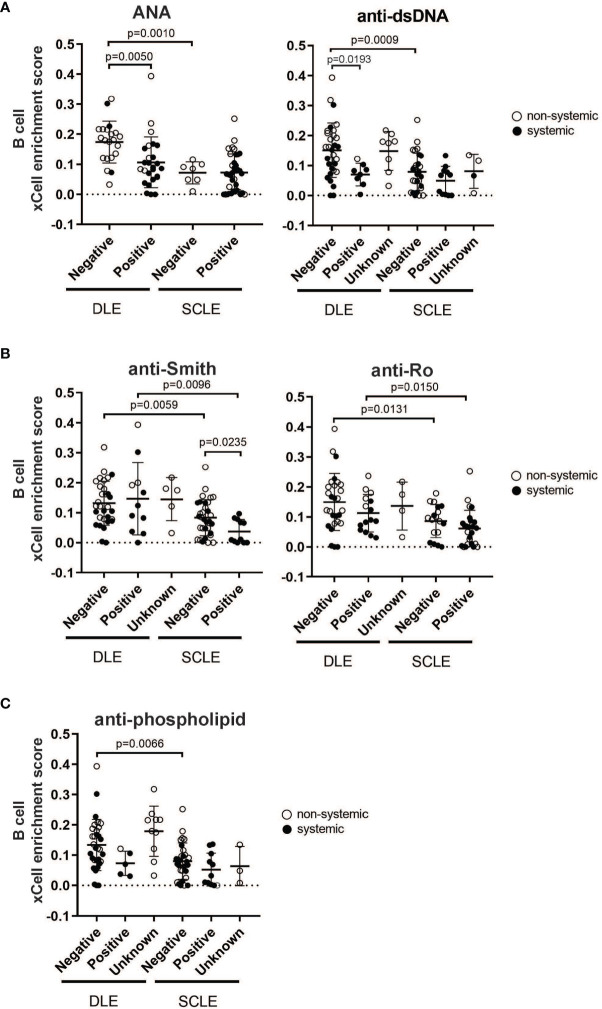
The relationship between skin B cell enrichment and the presence of circulating SLE autoantibodies in patients with DLE or SCLE. Patients with active DLE or SCLE skin lesions were stratified by the presence (positive) or absence (negative) of SLE autoantibodies at the time of biopsy. The patients in which the status of a particular autoantibody was not known at the time of biopsy were classified as “unknown”. **(A)** ANA and anti-dsDNA. **(B)** Anti-Smith and anti-Ro. **(C)** Anti-phospholipid. Comparisons were made *via* unpaired Students’ t-test.

Given that our data show that a prominent skin B cell gene signature is most pronounced in DLE lesions with a lack of underlying SLE, we sought to explore whether there is a relationship between skin B cell enrichment in lesional skin and the presence of peripheral lupus autoantibodies. We thus examined the lesional skin xCell B cell enrichment scores of DLE or SCLE patients subsetted by the presence of absence of key diagnostic lupus autoantibodies at the time of skin biopsy (**Figure 4**). As expected, DLE or SCLE patients with systemic disease were overall more likely to test positive for lupus antibodies than patients without systemic disease (**Figure 4**). Surprisingly, however, anti-nuclear antibody (ANA) negative DLE patients still demonstrated elevations in their cutaneous B cell signatures when compared to ANA+ DLE patients (**Figure 4A**). This was also true when the comparisons were made between anti-dsDNA- versus anti-dsDNA+ patients (**Figure 4A**). No differences were noted between ANA or anti-dsDNA positivity and B cell signatures in SCLE biopsies (**Figure 4A**). Interestingly, there was no difference between B cell enrichment score in DLE lesional skin for anti-Smith, anti-Ro, and anti-phospholipid antibodies (**Figures 4B, C**). In SCLE patients, only those positive for anti-Smith antibodies had significantly lower lesional skin B cell enrichment scores (**Figure 4B**, p=0.0235). Furthermore, we observed that skin B cell enrichment scores remained significantly higher in DLE lesions compared to SCLE lesions among patients who tested negative for ANA (p=0.0010) or anti-dsDNA (p=0.0009) (**Figure 4A**), anti-Smith (p=0.0059) or anti-Ro (p=0.0131) (**Figure 4B**), or anti-phospholipid antibodies (p=0.0066) (**Figure 4C**). Taken together, these data show that patients with DLE have elevated cutaneous B cell signatures without a concurrent subsequent rise in peripheral autoantibodies. This suggests that the function of B cells in CLE lesions may go beyond generation of antibody secreting cells or that cutaneously-produced antibodies either do not reach circulation or are against antigens that are not tested for on routine clinical testing.

### DLE Lesions Exhibit Higher Skin B Cell Numbers Compared to SCLE or ACLE

**Figure 5:**
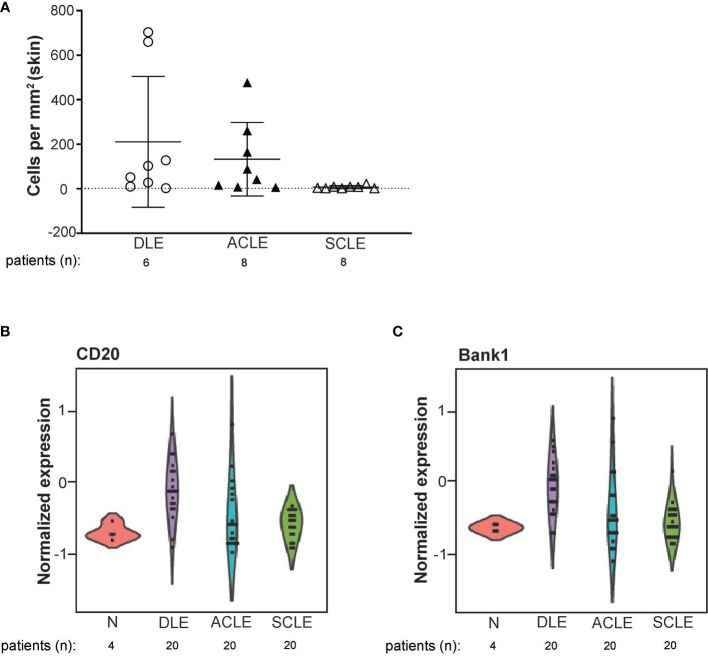
B cell quantification by tissue CyTOF and gene expression in lesional skin from ACLE, SCLE, and DLE patients. **(A)** The number of B cells numbers per millimeter of skin were quantified in SCLE (n = 8), ACLE (n = 8), and DLE (n = 8) lesions. Normalized gene expression of **(B)** CD20, and **(C)** Bank1 from SCLE (n = 20), ACLE (n = 20), and DLE (n = 20) lesional skin and normal healthy control skin (N) (n = 4).

To validate the transcriptional data and to expand our investigation to ACLE, immune cell populations were enumerated in lesional skin biopsies from patients with DLE, SCLE, or ACLE lesions by tissue CyTOF using a 16-antibody panel. While most immune cell populations, except for natural killer (NK) cells, neutrophils, and plasmablasts, were detectible in CLE lesions (**Supplementary Figure 3**), increased B cell numbers were only seen in DLE and ACLE lesions but not in SCLE (**Figure 5A**). This coincides with the skewing of certain B cell related genes in DLE>ACLE>SCLE lesions (**Figures 5B, C**).

**Figure 6:**
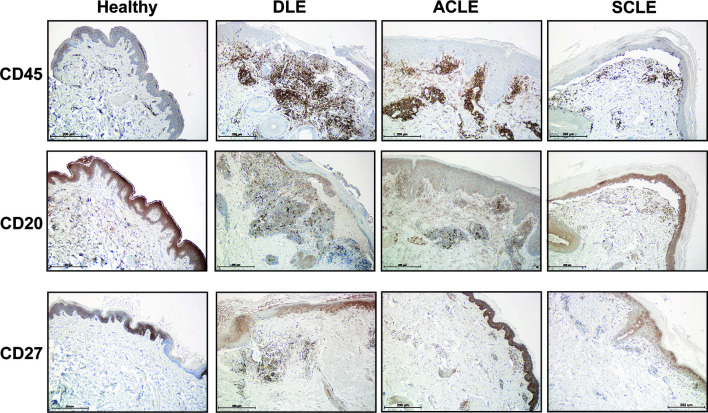
Immunohistochemistry staining for total immune cells and B cell subsets in lesional skin from healthy controls or patients with DLE, ACLE, or SCLE. Formalin-fixed paraffin embedded tissue sections from skin were stained for CD45+ total leukocytes, CD20+ B cells, and CD27+ mature B cells. Representative images from 3-5 patients of each subtype are shown at 100X magnification with a scale bar of 200 μm.

We then sought to confirm the distinct differences of a skin B cell signature across CLE subtypes *via* immunohistochemistry. Skin sections from ACLE, SCLE, and DLE lesions were probed with a pan-leukocyte marker (CD45), a broad B cell marker (CD20), and a marker for mature B cells (CD27) by immunohistochemistry (**Figure 6**). While an increase in CD45+ immune cells was observed in the skin of patents with CLE, we observed the highest infiltration of CD20+ B cells and CD27+ mature B cells in DLE lesional skin, followed by ACLE lesions, and SCLE lesional skin harbored the lowest infiltration of these immune cells among these CLE subtypes (**Figure 6** and **Supplementary Figure 4**). Taken together, these data reveal a gradient of B cell numbers and associated B cell marker gene expression with the highest in DLE and the lowest in SCLE.

## Discussion

The cellular and molecular basis of disease heterogeneity in CLE and the variability in which systemic lupus erythematosus (SLE) occurs in patients with different CLE subtypes remain important research objectives to understand lupus pathogenesis and develop precision therapies. To that end, we explored the transcriptional and cellular phenotypes of skin biopsies from patients with ACLE, SCLE, and DLE. As expected, we observed that interferon (IFN) genes were globally upregulated in both DLE and SCLE and therefore did not discriminate between these subtypes of disease. However, we subsequently identified a B cell gene signature in CLE skin that did indeed distinguish DLE from ACLE and SCLE, and this CLE-associated gene signature was highest in DLE lesions without associated systemic disease. These data indicate that while type I IFNs are known to contribute to the recruitment and activation of B cells in autoimmune disease (25–27), they may not be critical drivers in the differential recruitment of B cells observed in DLE skin.

Autoimmune responses and the contribution of B cells in SLE pathogenesis are well described, yet a role for skin-associated B cells in CLE is less apparent. There is considerable interest in the development of murine models to explore the contribution of B cells and other immune cell populations in the skin to cutaneous lupus pathogenesis (28, 29) and studies in the role of B cells in CLE patients are emerging. Indeed, our data suggest that while DLE is more likely to occur without underlying SLE compared to ACLE or SCLE (2, 5, 15), we detected a higher B cell signature in DLE lesions in patients without SLE that does not reflect peripheral autoantibody status. Our data mirror a previous study by Magro et al. that reported robust CD20 staining in 14/18 DLE lesions and only moderate CD20 staining in 9 SLE lesional biopsies (classification of biopsy subtype was not provided for SLE patients) (30). A more recent study that explored peripheral B cells in CCLE patients found that patients that lack systemic disease share peripheral B cell abnormalities with SLE patients (11). However, tissue-specific B cell responses in the skin of patients were not examined.

Thus, our data suggest that understanding tissue-specific B cell responses may be important for disease phenotype and possibly for predicting medication responses. Indeed, some small studies have suggested that SLE patients with refractory DLE respond better than SLE patients with SCLE to B cell depleting therapy (31). Another large study reported on 82 patients with SLE all of whom were treated with Rituximab (32). Importantly, no CLE without SLE patients were in this study. In this analysis, no DLE+SLE patients responded to Rituximab, yet of the ACLE patients that responded, negative anti-RNP and negative anti-Ro antibodies were associated with better response (32). Thus, we would propose that based on our data, DLE patients without SLE, especially those without a positive ANA, may be an important patient group to study for the effects of B cell depletion. Further clinical studies should address the benefit of B cell targeted therapy in isolated, refractory DLE.

Our study has several limitations. First, our study was performed retrospectively on archived patient samples. While this allows us to analyze a larger number of samples, we are limited in the clinical data and long-term follow-up that we were able to collect. Secondly, our discovery cohort did not include ACLE patients secondary to the design of that initial work. Future work should explore changes in skin B cell signatures over time in longitudinally collected patient samples and should include additional phenotyping studies to understand the role of B cells in the skin, whether they are contributing to antibody secretion, and whether their depletion can be a viable therapy in the right subset of patients.

In summary, we have identified a transcriptional B cell signature that is highest in DLE>ACLE>SCLE patients and that is most prominent when the CLE lesions occur without associated SLE. This was validated by immunostaining for both naïve and memory B cell populations in lesional skin. Interestingly, patients with skin lesions and positive autoantibodies tend to have a lower B cell enrichment score in the skin. This data has important implications for trial design for patients with isolated CLE, as treatment options for refractory CLE without SLE are limited. Further study into the role of B cells, the recruitment and differentiation in lesional skin, and the types of antibody secreting cells present will further enhance our ability to diagnose and treat CLE.

## Data Availability Statement

The original contributions presented in the study are publicly available. This data can be found here: https://www.ncbi.nlm.nih.gov/geo/ under the accession numbers GSE184989 and GSE81071 (https://www.ncbi.nlm.nih.gov/geo/).

## Ethics Statement

The studies involving human participants were reviewed and approved by IRBMED at University of Michigan. Written informed consent for participation was not required for this study in accordance with the national legislation and the institutional requirements.

## Author Contributions

Design of the study was performed by LA-C, JMK, JG, and CB. Sample and clinical data collection was performed by SL, RN, JS, and EM. Conducting experiments and data acquirement were done by LA-C, SR, CY, RN, LL, TR, and FW. Data and bioinformatics analyses were performed by LA-C, LT, SE, and CB. And interpretation of data and writing of drafts were performed by LA-C, LT, SE, JG, JMK, and CB. All authors contributed to the article and approved the submitted version.

## Funding

This study was funded by the National Institute of Arthritis and Musculoskeletal and Skin Diseases through awards R03AR072107, K24AR076975, and R01AR071384 (to JMK), and P30AR079374, and R01 AI130025 (to JG). Additional support was received from the Taubman Institute Innovative Projects Program (JMK, JG, and FW), and Dermatology Foundation (to JG). Some data in the validation cohort was also collected through support from Celgene to JMK and JG. Tissue CyTOF work was supported by NIH Office of the Director through award S10-OD020053 (to FW) and National Cancer Institute through award P30-CA046592 (to FW), and National Science Foundation through award 1653611 (to FW). In addition, LA-C was supported by grant number UL1TR002240 from the National Center for Advancing Translational Sciences (NCATS). We thank the support of George M. O’Brien Michigan Kidney Translational Research Core Center (P30DK081943).

## Conflict of Interest

JMK has received Grant support from Q32 Bio, Celgene/BMS, Ventus Therapeutics, and Janssen. JG has received Grant support from Celgene/BMS, Janssen, Eli Lilly, and Almirall. JMK has served on advisory boards for AstraZeneca, Eli Lilly, GlaxoSmithKline, Bristol Myers Squibb, Avion Pharmaceuticals, Provention Bio, Aurinia Pharmaceuticals, Ventus Therapeutics, and Boehringer Ingelheim. JG has served on advisory boards for AstraZeneca, Sanofi, Eli Lilly, Boehringer Ingelheim, Novartis, Janssen, Almirall, BMS.

The remaining authors declare that the research was conducted in the absence of any commercial or financial relationships that could be construed as a potential conflict of interest.

## Publisher’s Note

All claims expressed in this article are solely those of the authors and do not necessarily represent those of their affiliated organizations, or those of the publisher, the editors and the reviewers. Any product that may be evaluated in this article, or claim that may be made by its manufacturer, is not guaranteed or endorsed by the publisher.

## Supplementary Material

Supplementary Figure 1 Weighted correlation network analysis of DLE versus SCLE status and associated cyan and the IFN-score associated yellow modules in the validation cohort. **(A)** A total of 27 of the 32 Cyan module associated genes from the discovery cohort were also significantly higher in DLE compared to SCLE and ACLE, confirming the association of the cyan module with the DLE versus SCLE status (n = 20 per CLE subtype). **(B)** A total of 559 from the 746 yellow module associated genes from the discovery cohort also significantly correlated with the IFN score in the validation cohort (p-value < 0.0001).

Supplementary Figure 2 Cell type enrichment analysis using xCell tool, on both the discovery and the validation cohorts. Heatmap of relevant cell types representing average xCell score for each CLE disease compared to controls. Comparisons were made *via* unpaired Students’ t-test. Detailed summary statistics are presented in **Supplementary Table 4**. The asterisks represent the statistically significant changes in DLE compared to SCLE or ACLE compared to SCLE (p-value < 0.05).

Supplementary Figure 3 Immune cell quantification by tissue CyTOF and gene expression in lesional skin from ACLE, SCLE, and DLE patients. Immune cell populations in the skin were quantified in SCLE, ACLE, and DLE lesions.

Supplementary Figure 4 Immunohistochemistry staining for B cell subsets in lesional skin from additional patients with DLE, ACLE, or SCLE. Formalin-fixed paraffin embedded tissue sections from skin were stained for CD20+ B cells and CD27+ mature B cells (n = 3 patients per CLE subtype). Representative images are shown at 100X magnification with a scale bar of 200 μm.

Supplementary Table 2 Ingenuity pathway analysis from the 32 WGCNA cyan module genes: regulated canonical pathways. B cell-related pathways are highlighted in bold.

## References

[1] Lipsky PE. Systemic Lupus Erythematosus: An Autoimmune Disease of B Cell Hyperactivity. Nat Immunol (2001) 2(9):764–6. doi: 10.1038/ni0901-764 11526379

[2] Garelli CJ Refat MA Nanaware PP Ramirez-Ortiz ZG Rashighi M Richmond JM. Current Insights in Cutaneous Lupus Erythematosus Immunopathogenesis. Front Immunol (2020) 11:1353. doi: 10.3389/fimmu.2020.01353 32714331 PMC7343764

[3] Stannard JN Kahlenberg JM. Cutaneous Lupus Erythematosus: Updates on Pathogenesis and Associations With Systemic Lupus. Curr Opin Rheumatol (2016) 28(5):453–9. doi: 10.1097/BOR.0000000000000308 PMC4965280 27270345

[4] Okon LG Werth VP. Cutaneous Lupus Erythematosus: Diagnosis and Treatment. Best Pract Res Clin Rheumatol (2013) 27(3):391–404. doi: 10.1016/j.berh.2013.07.008 24238695 PMC3927537

[5] Grönhagen CM Fored CM Granath F Nyberg F. Cutaneous Lupus Erythematosus and the Association With Systemic Lupus Erythematosus: A Population-Based Cohort of 1088 Patients in Sweden. Br J Dermatol (2011) 164(6):1335–41. doi: 10.1111/j.1365-2133.2011.10272.x 21574972

[6] Baechler EC Batliwalla FM Karypis G Gaffney PM Ortmann WA Espe KJ. Interferon-Inducible Gene Expression Signature in Peripheral Blood Cells of Patients With Severe Lupus. Proc Natl Acad Sci U S A (2003) 100(5):2610–5. doi: 10.1073/pnas.0337679100 PMC151388 12604793

[7] Bennett L Palucka AK Arce E Cantrell V Borvak J Banchereau J. Interferon and Granulopoiesis Signatures in Systemic Lupus Erythematosus Blood. J Exp Med (2003) 197(6):711–23. doi: 10.1084/jem.20021553 PMC2193846 12642603

[8] Crow MK. Type I Interferon in the Pathogenesis of Lupus. J Immunol (2014) 192(12):5459–68. doi: 10.4049/jimmunol.1002795 PMC4083591 24907379

[9] Sarkar MK Hile GA Tsoi LC Xing X Liu J Liang Y. Photosensitivity and Type I IFN Responses in Cutaneous Lupus are Driven by Epidermal-Derived Interferon Kappa. Ann Rheum Dis (2018) 77(11):1653–64. doi: 10.1136/annrheumdis-2018-213197 PMC6185784 30021804

[10] Zhu JL Tran LT Smith M Zheng F Cai L James JA. Modular Gene Analysis Reveals Distinct Molecular Signatures for Subsets of Patients With Cutaneous Lupus Erythematosus. Br J Dermatol (2021) 185(3):563–72. doi: 10.1111/bjd.19800 PMC8255330 33400293

[11] Jenks SA Wei C Bugrovsky R Hill A Wang X Rossi FM. B Cell Subset Composition Segments Clinically and Serologically Distinct Groups in Chronic Cutaneous Lupus Erythematosus. Ann Rheum Dis (2021). doi: 10.1136/annrheumdis-2021-220349 PMC8906255 34083207

[12] Kil LP Hendriks RW. Aberrant B Cell Selection and Activation in Systemic Lupus Erythematosus. Int Rev Immunol (2013) 32(4):445–70. doi: 10.3109/08830185.2013.786712 23768157

[13] Berthier CC Tsoi LC Reed TJ Stannard JN Myers EM Namas R. Molecular Profiling of Cutaneous Lupus Lesions Identifies Subgroups Distinct From Clinical Phenotypes. J Clin Med (2019) 8(8). doi: 10.3390/jcm8081244 PMC6723404 31426521

[14] Ko WC Li L Young TR McLean-Mandell RE Deng AC Vanguri VK. Gene Expression Profiling in Skin Reveals Strong Similarities Between Subacute and Chronic Cutaneous Lupus That are Distinct From Lupus Nephritis. J Invest Dermatol (2021). doi: 10.1016/j.jid.2021.04.030 34153327

[15] Vera-Recabarren MA García-Carrasco M Ramos-Casals M Herrero C. Comparative Analysis of Subacute Cutaneous Lupus Erythematosus and Chronic Cutaneous Lupus Erythematosus: Clinical and Immunological Study of 270 Patients. Br J Dermatol (2010) 162(1):91–101. doi: 10.1111/j.1365-2133.2009.09472.x 19785596

[16] Maz MP Michelle Kahlenberg J. Cutaneous and Systemic Connections in Lupus. Curr Opin Rheumatol (2020) 32(6):583–9. doi: 10.1097/BOR.0000000000000739 PMC8000781 32826479

[17] Hochberg MC. Updating the American College of Rheumatology Revised Criteria for the Classification of Systemic Lupus Erythematosus. Arthritis Rheum (1997) 40(9):1725. doi: 10.1002/art.1780400928 9324032

[18] Irizarry RA Hobbs B Collin F Beazer-Barclay YD Antonellis KJ Scherf U. Exploration, Normalization, and Summaries of High Density Oligonucleotide Array Probe Level Data. Biostatistics (2003) 4(2):249–64. doi: 10.1093/biostatistics/4.2.249 12925520

[19] Leek JT Johnson WE Parker HS Jaffe AE Storey JD. The Sva Package for Removing Batch Effects and Other Unwanted Variation in High-Throughput Experiments. Bioinformatics (2012) 28(6):882–3. doi: 10.1093/bioinformatics/bts034 PMC3307112 22257669

[20] Law CW Chen Y Shi W Smyth GK. Voom: Precision Weights Unlock Linear Model Analysis Tools for RNA-Seq Read Counts. Genome Biol (2014) 15(2):R29. doi: 10.1186/gb-2014-15-2-r29 24485249 PMC4053721

[21] Ritchie ME Phipson B Wu D Hu Y Law CW Shi W. Limma Powers Differential Expression Analyses for RNA-Sequencing and Microarray Studies. Nucleic Acids Res (2015) 43(7):e47. doi: 10.1093/nar/gkv007 25605792 PMC4402510

[22] Langfelder P Horvath S. WGCNA: An R Package for Weighted Correlation Network Analysis. BMC Bioinformatics (2008) 9:559. doi: 10.1186/1471-2105-9-559 19114008 PMC2631488

[23] Aran D Hu Z Butte AJ. Xcell: Digitally Portraying the Tissue Cellular Heterogeneity Landscape. Genome Biol (2017) 18(1):220. doi: 10.1186/s13059-017-1349-1 29141660 PMC5688663

[24] Gudjonsson JE Tsoi LC Ma F Billi AC van Straalen KR Vossen ARJV. Contribution of Plasma Cells and B Cells to Hidradenitis Suppurativa Pathogenesis. JCI Insight (2020) 5(19). doi: 10.1172/jci.insight.139930 PMC7566715 32853177

[25] Kiefer K Oropallo MA Cancro MP Marshak-Rothstein A. Role of Type I Interferons in the Activation of Autoreactive B Cells. Immunol Cell Biol (2012) 90(5):498–504. doi: 10.1038/icb.2012.10 22430248 PMC3701256

[26] Keller EJ Patel NB Patt M Nguyen JK Jørgensen TN. Partial Protection From Lupus-Like Disease by B-Cell Specific Type I Interferon Receptor Deficiency. Front Immunol (2020) 11:616064. doi: 10.3389/fimmu.2020.616064 33488628 PMC7821742

[27] Liu M Guo Q Wu C Sterlin D Goswami S Zhang Y. Type I Interferons Promote the Survival and Proinflammatory Properties of Transitional B Cells in Systemic Lupus Erythematosus Patients. Cell Mol Immunol (2019) 16(4):367–79. doi: 10.1038/s41423-018-0010-6 PMC6461980 29563616

[28] Zhou S Li Q Zhao M Lu L Wu H Lu Q. A Novel Humanized Cutaneous Lupus Erythematosus Mouse Model Mediated by IL-21-Induced Age-Associated B Cells. J Autoimmun (2021) 123:102686. doi: 10.1016/j.jaut.2021.102686 34325305

[29] Mande P Zirak B Ko WC Taravati K Bride KL Brodeur TY. Fas Ligand Promotes an Inducible TLR-Dependent Model of Cutaneous Lupus-Like Inflammation. J Clin Invest (2018) 128(7):2966–78. doi: 10.1172/JCI98219 PMC6025993 29889098

[30] Magro CM Segal JP Crowson AN Chadwick P. The Phenotypic Profile of Dermatomyositis and Lupus Erythematosus: A Comparative Analysis. J Cutan Pathol (2010) 37(6):659–71. doi: 10.1111/j.1600-0560.2009.01443.x 19891658

[31] Quelhas da Costa R Aguirre-Alastuey ME Isenberg DA Saracino AM. Assessment of Response to B-Cell Depletion Using Rituximab in Cutaneous Lupus Erythematosus. JAMA Dermatol (2018) 154(12):1432–40. doi: 10.1001/jamadermatol.2018.3793 PMC6583321 30383114

[32] Vital EM Wittmann M Edward S Md Yusof MY MacIver H Pease CT. Brief Report: Responses to Rituximab Suggest B Cell-Independent Inflammation in Cutaneous Systemic Lupus Erythematosus. Arthritis Rheumatol (2015) 67(6):1586–91. doi: 10.1002/art.39085 25707733

