# Supplementary figures and images for: B Cell Signatures Distinguish Cutaneous Lupus Erythematosus Subtypes and the Presence of Systemic Disease Activity
Source: Front Immunol. 2021 Nov 19;12:775353. doi: 10.3389/fimmu.2021.775353 (PMC8640489; doi:10.3389/fimmu.2021.775353)

# Supplemental Figure 1

A.

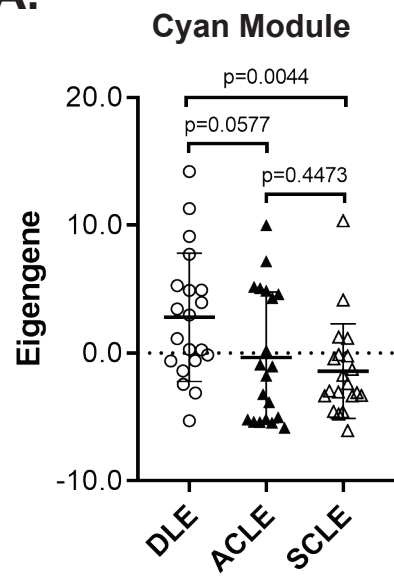

B.

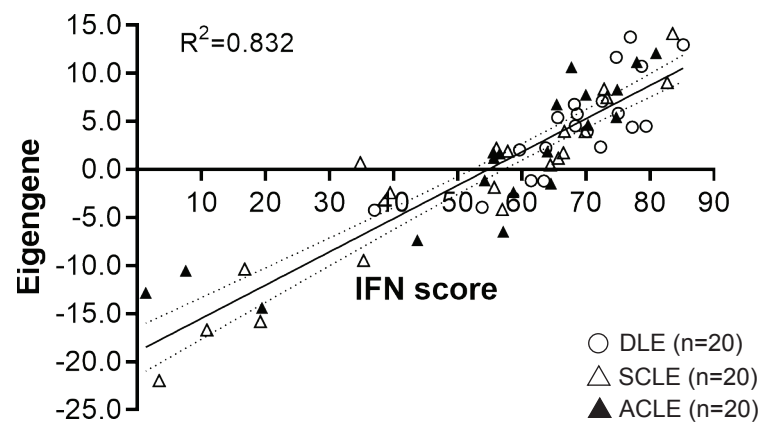

Supplement: Supplementary Figure 1 — Weighted correlation network analysis of DLE versus SCLE status and associated cyan and the IFN-score associated yellow modules in the validation cohort. (A) A total of 27 of the 32 Cyan module associated genes from the discovery cohort were also significantly higher in DLE compared to SCLE and ACLE, confirming the association of the cyan module with the DLE versus SCLE status (n = 20 per CLE subtype). (B) A total of 559 from the 746 yellow module associated genes from the discovery cohort also significantly correlated with the IFN score in the validation cohort (p-value < 0.0001). [file DataSheet_1.pdf]

# Supplemental Figure 2

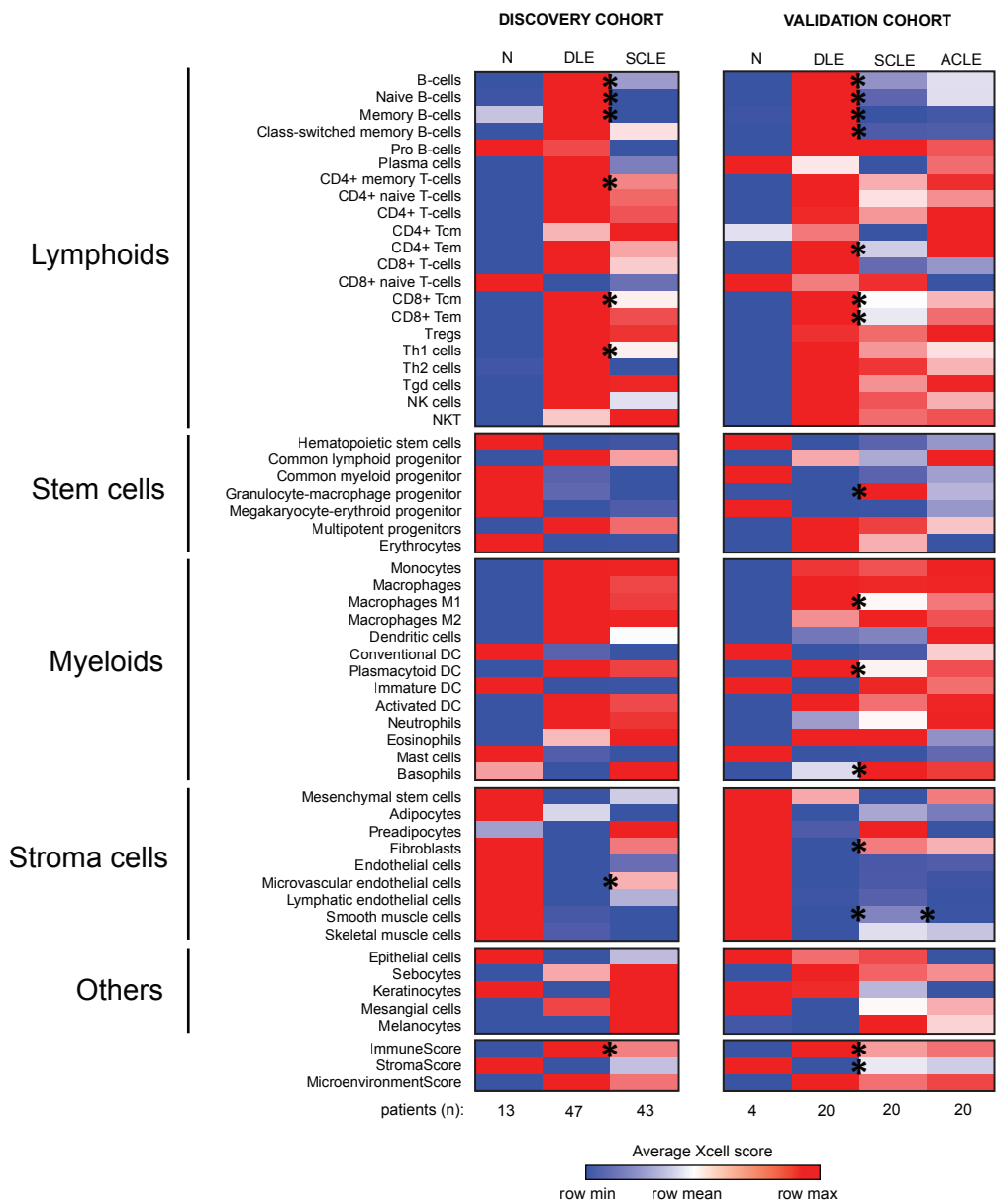

Supplement: Supplementary Figure 2 — Cell type enrichment analysis using xCell tool, on both the discovery and the validation cohorts. Heatmap of relevant cell types representing average xCell score for each CLE disease compared to controls. Comparisons were made via unpaired Students’ t-test. Detailed summary statistics are presented in Supplementary Table 4 . The asterisks represent the statistically significant changes in DLE compared to SCLE or ACLE compared to SCLE (p-value < 0.05). [file DataSheet_2.pdf]

Supplemental Figure 3

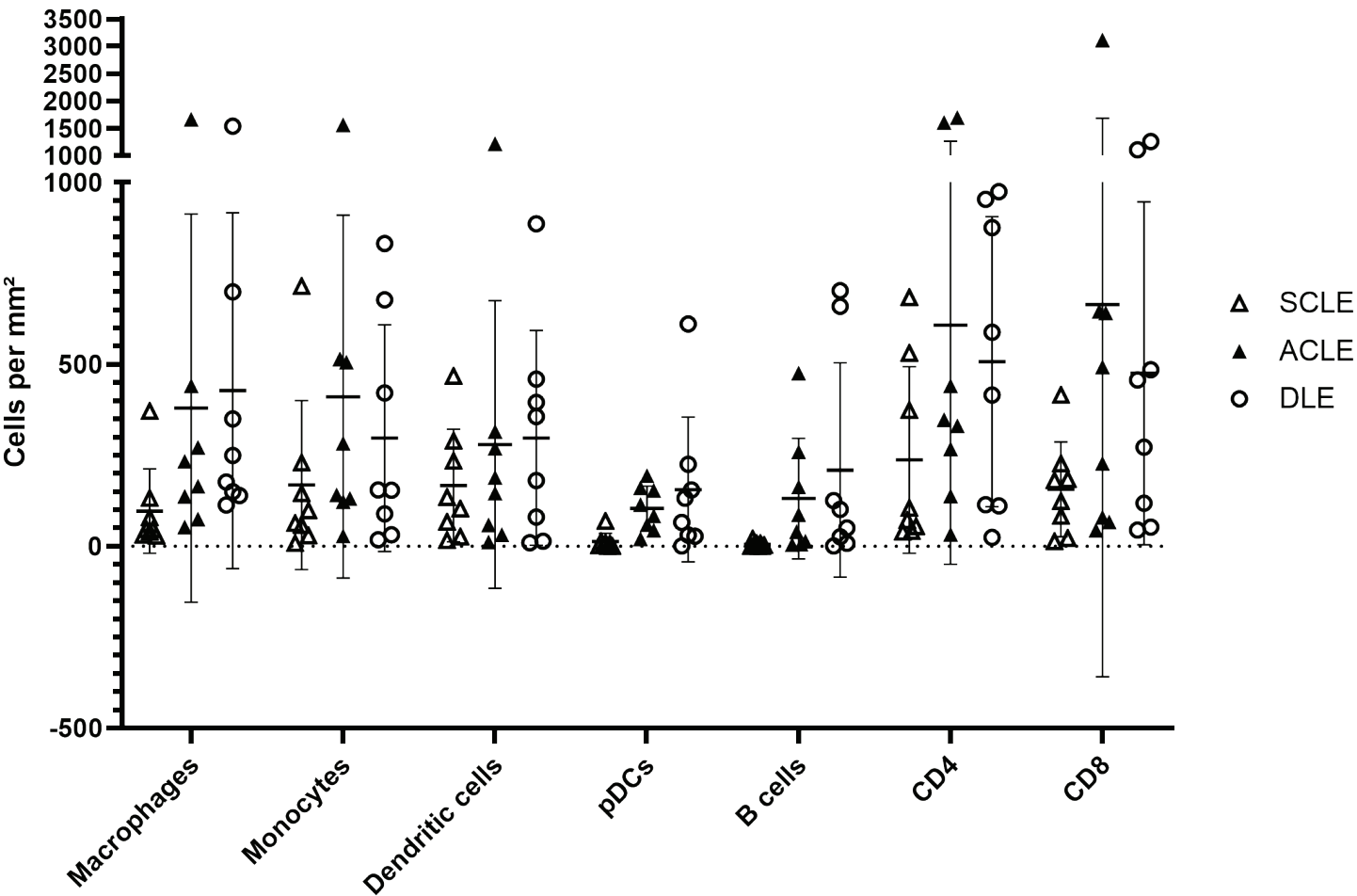

Supplement: Supplementary Figure 3 — Immune cell quantification by tissue CyTOF and gene expression in lesional skin from ACLE, SCLE, and DLE patients. Immune cell populations in the skin were quantified in SCLE, ACLE, and DLE lesions. [file DataSheet_3.pdf]

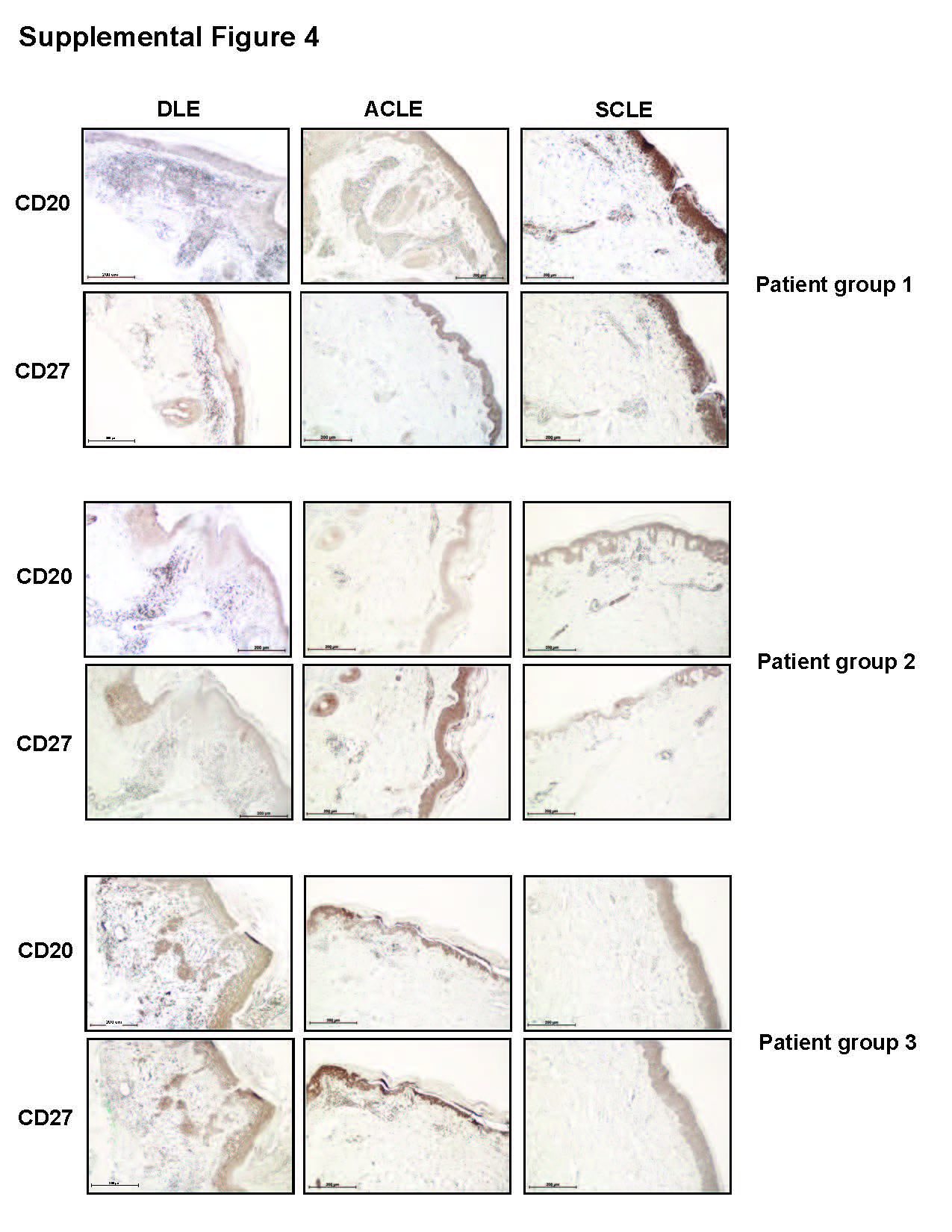

Supplement: Supplementary Figure 4 — Immunohistochemistry staining for B cell subsets in lesional skin from additional patients with DLE, ACLE, or SCLE. Formalin-fixed paraffin embedded tissue sections from skin were stained for CD20+ B cells and CD27+ mature B cells (n = 3 patients per CLE subtype). Representative images are shown at 100X magnification with a scale bar of 200 μm. [file Image_4.jpeg]
