# Supplementary material for: B Cell Signatures Distinguish Cutaneous Lupus Erythematosus Subtypes and the Presence of Systemic Disease Activity
Source: Front Immunol. 2021 Nov 19;12:775353. doi: 10.3389/fimmu.2021.775353 (PMC8640489; doi:10.3389/fimmu.2021.775353)
Supplement: Supplementary file 5 [file Table_1.pdf]

Supplemental Table 1. Summary characteristics of lupus patients included in the CLE discovery cohort (published in Berthier & Tsoi, 2019) and the validation cohort. Clinical data were not available for 9 DLE patients, 12 SCLE patients, and 1 ACLE patient in the validation cohort.

DISCOVERY COHORT	DLE (n=47)	SCLE (n=43)
Mean Age (years±SEM)	45.2 (2.0)	47.2 (2.7)
Gender (% Female)	78.7	83.7
Ethnicity (% African-American)	40.4	4.7
Body Mass Index (±SEM)	28.0 (0.9)	27.6 (1.2)
SLEDAI (±SEM)	7.1 (0.8)	9.3 (1.2)
CLASI Activity (±SEM)	6.4 (0.5)	8.2 (0.6)
CLASI Damage (±SEM)	4.2 (0.7)	0.7 (0.3)
% ≥4 ACR criteria for SLE within 3 years of biopsy	46.8	55.8
Lupus nephritis (%)	4.3	11.6
Positive anti-Smith antibodies (%)	23.4	25.6
Positive anti- Ro antibodies (%)	31.9	58.1
Positive antiphospholipid antibodies (%)	10.6	25.6
dsDNA titer (±SEM)	9.6 (2.8)	35.0 (13.4)
IFN score (±SEM)	19.3 (0.2)	15.8 (0.2)
Medications (% on drug at time of biopsy)		
Oral Prednisone <10 mg daily	8.5	4.7
Oral Prednisone >10 mg daily	10.6	23.3
Mycophenolate Mofetil	2.1	11.6
Antimalarials	40.4	44.2
Methotrexate	2.1	0

VALIDATION COHORT	DLE (n=10)	SCLE (n=9)	ACLE (n=19)
Mean Age (years±SEM)	45.2 (6.3)	65.2 (4.6)	44.4 (3.4)
Gender (% Female)	80.0	88.9	89.5
Ethnicity (% African-American)	30.0	0	36.8
Body Mass Index (±SEM)	24.8 (1.3)	25.5 (1.5)	29.6 (2.0)
SLEDAI (±SEM)	13.3 (0.7)[a]	8.0 (3.1)[a]	9.9 (2.1)[b]
CLASI Activity (±SEM)	10.3 (1.6)	15.4 (2.4)	10.7 (2.2)
CLASI Damage (±SEM)	3.8 (1.2)	2.0 (1.4)	2.5 (1.1)
% ≥4 ACR criteria for SLE within 3 years of biopsy	30.0	33.3	57.9
Lupus nephritis (%)	0.0	0.0	10.5
Positive anti-Smith antibodies (%)	0.0[c]	12.5[c]	15.8
Positive anti- Ro antibodies (%)	50.0[c]	77.8	52.6
Positive antiphospholipid antibodies (%)	0.0[d]	0.0[a]	35.7[e]
dsDNA titer (±SEM)	166.0 (21.1)[f]	35.5 (35.5)[g]	252.0 (61.0)
IFN score (±SEM)*	68.6 (5.3)	51.6 (12.1)	56.0 (11.1)
Medications (% on drug at time of biopsy)			
Oral Prednisone <10 mg daily	10.0	11.1	10.5

Oral Prednisone >10 mg daily	20.0	22.2	26.3
Mycophenolate Mofetil	10.0	0.0	15.8
Antimalarials	10.0	33.3	36.8
Methotrexate	0.0	11.1	5.3

*IFN score was calculated from gene expression data from all 20 DLE, 20 SCLE and 20 ACLE patients. [a]Data were available for 3 patients. [b]Data were available for 11 patients. [C]Data were available for 8 patients. [d]Data were available for 4 patients. [e]Data were available for 14 patients. [f]Data were available for 9 patients. [g]Data were available for 5 patients.
