# Supplementary material for: B Cell Signatures Distinguish Cutaneous Lupus Erythematosus Subtypes and the Presence of Systemic Disease Activity
Source: Front Immunol. 2021 Nov 19;12:775353. doi: 10.3389/fimmu.2021.775353 (PMC8640489; doi:10.3389/fimmu.2021.775353)
Supplement: Supplementary Table 2 — Ingenuity pathway analysis from the 32 WGCNA cyan module genes: regulated canonical pathways. B cell-related pathways are highlighted in bold. [file Table_2.pdf]

Supplemental Table 2

Pathway	p-value
**B Cell Receptor Signaling**	6.31E-33
IL-15 Signaling	2.51E-32
**Systemic Lupus Erythematosus in B Cell Signaling Pathway**	1.58E-31
Communication between Innate and Adaptive Immune Cells	3.98E-31
Primary Immunodeficiency Signaling	4.47E-09
Role of Macrophages, Fibroblasts and Endothelial Cells in Rheumatoid Arthritis	4.79E-04
Systemic Lupus Erythematosus Signaling	6.17E-04
Phospholipase C Signaling	8.91E-04
Hematopoiesis from Pluripotent Stem Cells	1.51E-03
Autoimmune Thyroid Disease Signaling	1.66E-03
Allograft Rejection Signaling	2.09E-03
Dendritic Cell Maturation	4.27E-03
Phagosome Formation	7.41E-03
Altered T Cell and B Cell Signaling in Rheumatoid Arthritis	1.82E-02
April Mediated Signaling	4.68E-02
**B Cell Activating Factor Signaling**	4.79E-02
**B Cell Development**	4.79E-02
