# Supplementary material for: B Cell Signatures Distinguish Cutaneous Lupus Erythematosus Subtypes and the Presence of Systemic Disease Activity
Source: Front Immunol. 2021 Nov 19;12:775353. doi: 10.3389/fimmu.2021.775353 (PMC8640489; doi:10.3389/fimmu.2021.775353)
Supplement: Supplementary file 7 [file Table_3.pdf]

Supplemental Table 3: List of the 746 genes associated with IFN score in the yellow module of the WGCNA analysis from the discovery cohort.

AAMP	CTBS	IFI27	OASL	SNORD4A
ABHD10	CTSL	IFI30	OAZ2	SNORD7
ACIN1	CTSLP2	IFI35	ODF3B	SNORD70
ACLY	CX3CL1	IFI44	OGFR	SNORD8
ACP2	CXCL1	IFI44L	OTUD4	SNRPC
ACTA2-AS1	CXCL10	IFI6	P2RY6	SNX2
ADA	CXCL11	IFIH1	PANK2	SOCS1
ADAR	CXCL16	IFIT1	PARP11	SP100
ADCY7	CXCR2P1	IFIT2	PARP12	SP110
ADD1	CXorf38	IFIT3	PARP14	SPATS2L
ADGRE5	CYBA	IFIT5	PARP9	SPI1
ADPRH	CYREN	IFITM1	PAX8	SRA1
ADRB2	CYTH1	IFITM3	PCNX1	SS18L2
AGAP3	CYTH4	IFITM4P	PDCD1LG2	ST3GAL1
AGK	DBNL	IGHM	PDIA4	ST3GAL5
AGO2	DCP2	IGHV3-30	PELO	STARD4
AGPAT3	DDIT3	IGHV3-7	PFKP	STAT1
AIM2	DDX10	IKBKE	PHF11	STAT2
AKIRIN1	DDX58	IL12RB2	PI4K2B	STAT6
AKT1	DDX60	IL15RA	PIK3AP1	STK10
AKT1S1	DDX60L	IL1B	PILRA	STK11
AKT2	DENND2D	IL6R	PLAAT2	STOM
ALAS1	DENND5A	INTS7	PLAAT4	STX4
ALG11	DGLUCY	IRF7	PLAC8	STXBP2
ALPK1	DHX58	IRF9	PLAUR	STYXL1
AMBRA1	DIABLO	ISG15	PLEKHB2	SULF1
AMMECR1	DIAPH1-AS1	ISG20	PLEKHO2	SUMO3
ANGPTL6	DIS3L2	ITGB7	PLK4	SYK
ANKFY1	DMAP1	ITPK1	PLSCR1	SYMPK
ANKRD22	DNAI3	JDP2	PML	SYNGR2
ANXA10	DNAJA1	JPT1	PNPO	TAP1
APOBEC3A	DNASE2	KCNE1	PNPT1	TAP2
APOBEC3D	DNM2	KCNQ5-IT1	POLDIP3	TAPBP
APOBEC3G	DNMT1	KCTD2	PPM1K	TCN2
APOBEC3H	DOCK8-AS1	KCTD5	PPP1R18	TCTA
APOC1	DOT1L	KDM4A-AS1	PPP6R1	TDO2
APOL1	DPM2	KHDC4	PPP6R2	TDRD7
APOL2	DRAM1	KIAA0040	PRAM1	TEC
APOL3	DRAP1	KIF21B	PRKCD	TEX48
APOL6	DTX3L	KLF16	PRKCE	TFEB
AQP9	DUSP10	KLF6	PRKD2	TGFB1
ARAP1	DUSP3	KLHDC7B	PRPF3	TGOLN2
ARF4	DUSP5	KRT6A	PRR13	TH2LCRR
ARFGAP1	DUSP6	KYNU	PRR14L	THEMIS2
ARHGAP4	DYNLT1	LAG3	PSD4	THOC7-AS1
ARHGDIA	ECT2	LAIR1	PSENEN	TIMMDC1

ARHGEF11	EDARADD	LAMP3	PSMB2	TK1
ARID1A	EDEM1	LAP3	PSMB8	TLE5
ARID5A	EFHD2	LASP1	PSMB8-AS1	TLK2
ARL14	EGFL6	LCN2	PSMB9	TLR2
ARL4C	EHBP1L1	LGALS17A	PSMD9	TLR3
ARPC1B	EHD4	LGALS3BP	PSME1	TLR7
ARPP19	EIF1AY	LILRB1	PSME2	TMEM140
ARRDC1	EIF2AK2	LILRB3	PSMF1	TMEM222
ASAP1-IT1	ELAVL1	LILRB4	PTAFR	TMEM268
ASB16-AS1	ELF4	LINC00487	PTGER2	TMEM60
ATP10A	ELK1	LINC01260	PTK2B	TNFAIP2
ATP13A2	ELMO2	LINC02316	PTOV1-AS1	TNFRSF10B
ATP6V1F	EMP3	LINS1	PTPN23	TNFRSF14
ATP8B4	ENO1	LIPG	PTPRE	TNFSF10
ATXN7	ENPP2	LLPH	PTTG1	TNFSF13B
B2M	EPB41L3	LMNB1	PUDP	TNFSF15
B4GALT5	EPSTI1	LOC100288175	QRSL1	TNIP1
BATF2	ETV7	LOC100288637	RAB33B	TNK2
BAX	EXOSC9	LOC101926887	RAB35	TPTE2P5
BCL2A1	FAM104A	LOC101927377	RAB39A	TRAF3
BCL3	FAM20A	LOC101927762	RAB8A	TRAFD1
BCL7B	FAM225A	LOC101929540	RAD23A	TRANK1
BIN1	FAM53B	LOC102724420	RAP1B	TRAPPC1
BIRC2	FAM83G	LOC284454	RAP1GAP2	TRAV19
BLVRA	FAS	LOC644135	RAP2B	TRBJ2-1
BRCA2	FAS-AS1	LPIN2	RARA	TRBJ2-2
BRIP1	FBXO5	LRRC25	RBCK1	TRDJ1
BRMS1	FBXO6	LRRC4	RBM14	TRDV1
BSDC1	FCER1G	LRRC42	RELA	TREX1
BST2	FCGR1B	LRRC59	RELB	TRIM14
BTN3A1	FCN1	LSP1	REXO4	TRIM21
BTN3A2	FFAR2	LY6E	RGL1	TRIM22
BUB1	FGFR1OP2	LY6E-DT	RHBDF2	TRIM25
BUD23	FIGNL1	LYN	RHOG	TRIM38
C1GALT1	FLJ42393	LYSMD2	RILPL1	TRIM4
C1orf74	FLVCR2	MAEA	RILPL2	TRIM5
C1QA	FMNL1	MAP2K1	RLIM	TRIM56
C1QB	FOSL1	MAP3K11	RNF114	TRIM69
C1QC	FPR1	MAP3K8	RNF138	TRMT11
C2	FPR2	MARK2	RNF19B	TRPC4AP
C2CD4B	FPR3	MASTL	RNF213	TTC38
C3AR1	FSCN1	MAX	RNF31	TTC5
C3orf38	FUOM	MBD1	RNF40	TTC7A
C5AR1	FUT4	MCFD2	RNU5F-1	TTLL4
C6orf62	FZR1	MCL1	RNU6-59P	TYMP
CADM3-AS1	GADD45B	MCM10	RPL13P5	TYROBP
CALM3	GALNT10	MCRIP1	RPL23AP7	UBA7
CALR	GBP1	MDK	RRM2B	UBALD1
CARD16	GBP1P1	MED15	RSAD2	UBALD2

CASP1	GBP2	MGAT1	RTP4	UBAP1
CASP10	GBP3	MILR1	RUBCN	UBE2L6
CBX6	GBP4	MIR1183	RUFY4	UHMK1
CC2D1B	GCH1	MIR142	RUNX2	UNC93B1
CCDC12	GCNT1	MIR3140	RUNX3	USF2
CCDC9	GCNT2	MIR3174	RXRB	USP15
CCL2	GFOD1	MIR4441	SAMD9	USP18
CCL3	GGA2	MIR4517	SAMD9L	USP28
CCL5	GLIS3	MIR548Q	SAMHD1	USP30-AS1
CCL7	GLT1D1	MIR643	SAT1	USP36
CCL8	GLUL	MKNK2	SBNO2	VAMP8
CCM2	GMIP	MLKL	SCAF1	VASP
CCND1	GMPR	MMP1	SCAMP3	VCPIP1
CCR1	GNB2	MOB3A	SCML4	VTRNA1-1
CCR5	GOLM1	MOB3C	SCO2	VTRNA1-3
CCRL2	GPD2	MPDU1	SDC3	WARS1
CD14	GPR18	MRPL44	SECTM1	WASIR2
CD180	GPX4	MTHFD2	SEMA4D	WDFY1
CD274	GREM1	MVP	SEPTIN9	WDTC1
CD38	GRINA	MX1	SERPINB1	WNT10A
CD47	GRK6	MX2	SERPING1	XAF1
CD68	GTF2F1	MYD88	SETD1B	XRN1
CD80	GTPBP1	MYDGF	SF1	XRRA1
CD82	GZMB	MYO9B	SH3BP1	YEATS2
CDC25B	H2BC12	N4BP1	SH3BP2	YIPF2
CDKN1B	H2BC14	NABP1	SHFL	YJU2
CEBPA	H2BC5	NAGK	SHKBP1	ZBP1
CFB	H3C11	NAMPT	SIGLEC14	ZBTB17
CH25H	H3C4	NANS	SIK3	ZC3H10
CHAD	H4C13	NAPA	SIRPB1	ZC3HAV1
CHEK1	HAVCR2	NBEAL2	SLC15A3	ZCCHC2
CHMP5	HCP5	NCF2	SLC31A2	ZFAND3
CHROMR	HDAC1	NDUFAF4	SLC35A4	ZFAT
CHST12	HDDC3	NECTIN2	SLC38A5	ZFP36L2
CIAO2A	HERC5	NFATC2IP	SLC45A4	ZFYVE26
CIAO2B	HERC6	NFKB1	SLFN12	ZMIZ2
CIB1	HESX1	NFKB2	SLFN5	ZNF189
CISH	HIRA	NFKBIA	SMCHD1	ZNF235
CKAP2L	HLA-A	NIPSNAP1	SMTNL1	ZNF296
CLCN7	HLA-B	NLRC5	SNAR-D	ZNF484
CLDN20	HLA-C	NMI	SNAR-G1	ZNF526
CLEC16A	HLA-DRB1	NNT-AS1	SNAR-G2	ZNF534
CLEC7A	HLA-E	NOD1	SNORA1	ZNF552
CLK3	HLA-F	NR1H2	SNORA11B	ZNF580
CMPK2	HLA-H	NRIP1	SNORA13	ZNF589
CMTR1	HMGXB3	NT5C3A	SNORA25	ZNF592
CNDP2	HNRNPF	NT5E	SNORA31	ZNF687
CNKSR1	HOXB2	NUB1	SNORA65	ZNF706
CNP	HSCB	NUCB1	SNORA74A	ZNF707

CNPY3	HSH2D	NUDCD1	SNORA74B	ZNFX1
COPS7A	HSP90AB4P	NUP210	SNORA9	ZRSR2
COQ10B	ICAM1	NUP62	SNORD116-14	
CPNE1	IDO1	OAS1	SNORD12	
CRAMP1	IER5	OAS2	SNORD15B	
CSF1	IFI16	OAS3	SNORD21	
