# Supplementary material for: B Cell Signatures Distinguish Cutaneous Lupus Erythematosus Subtypes and the Presence of Systemic Disease Activity
Source: Front Immunol. 2021 Nov 19;12:775353. doi: 10.3389/fimmu.2021.775353 (PMC8640489; doi:10.3389/fimmu.2021.775353)
Supplement: Supplementary file 8 [file Table_4.pdf]

Supplemental Table 4. xCell analysis results and statistics from the discovery and validation cohorts. Comparisons between study groups were made using two-sided unpaired Students' *t-test*. P-values<0.05 are highlighted in bold.

**DISCOVERY COHORT**							
**Cell type**	**Mean CTLS**	**STDEV CTLS**	**Mean DLE**	**STDEV DLE**	**Mean SCLE**	**STDEV SCLE**	**p-value (DLE vs SCLE)**
**B-cells**	0.0427	0.0492	0.1370	0.0845	0.0721	0.0588	**0.0001**
**naive B-cells**	0.0018	0.0065	0.0176	0.0323	0.0009	0.0040	**0.0011**
**Memory B-cells**	0.0388	0.0434	0.0626	0.0493	0.0238	0.0305	**0.0000**
Class-switched memory B-cells	0.0749	0.0741	0.1248	0.0810	0.1027	0.0747	0.1840
pro B-cells	0.0384	0.0506	0.0366	0.0350	0.0252	0.0326	0.1126
Plasma cells	0.0225	0.0191	0.0273	0.0414	0.0237	0.0356	0.6607
CD4+ memory T-cells	0.1201	0.0944	0.3187	0.1119	0.2666	0.1187	0.0349
CD4+ naive T-cells	0.0631	0.0560	0.0708	0.0567	0.0693	0.0568	0.8965
CD4+ T-cells	0.0788	0.0690	0.1770	0.0870	0.1615	0.0864	0.3993
CD4+ Tcm	0.0461	0.0630	0.0635	0.0634	0.0732	0.0720	0.4986
CD4+ Tem	0.0954	0.0998	0.2025	0.1036	0.1675	0.0977	0.1033
CD8+ T-cells	0.0568	0.0400	0.1039	0.0541	0.0851	0.0502	0.0911
CD8+ naive T-cells	0.1018	0.0750	0.0573	0.0538	0.0673	0.0570	0.3912
CD8+ Tcm	0.0578	0.0713	0.1201	0.0650	0.0911	0.0588	0.0295
CD8+ Tem	0.0557	0.0708	0.2015	0.0762	0.1799	0.0589	0.1398
Tregs	0.0324	0.0402	0.1121	0.0663	0.1052	0.0818	0.6569
**Th1 cells**	0.1367	0.1022	0.2459	0.1250	0.1951	0.1082	**0.0431**
Th2 cells	0.0183	0.0238	0.0288	0.0414	0.0176	0.0248	0.1284
Tgd cells	0.0820	0.0530	0.1310	0.0534	0.1289	0.0471	0.8426
NK cells	0.0438	0.0665	0.0682	0.0665	0.0546	0.0555	0.2956
NKT	0.2230	0.1382	0.2472	0.1682	0.2629	0.1673	0.6579
Hematopoietic stem cells	0.3848	0.0860	0.2242	0.1122	0.2339	0.1101	0.6785
Common lymphoid progenitor	0.1187	0.0637	0.1325	0.0507	0.1281	0.0554	0.6963
Common myeloid progenitor	0.0995	0.0524	0.0606	0.0496	0.0524	0.0508	0.4404
Granulocyte-macrophage progenitor	0.0928	0.0672	0.0490	0.0495	0.0383	0.0505	0.3127
Megakaryocyte-erythroid progenitor	0.0931	0.0772	0.0194	0.0367	0.0293	0.0428	0.2407
Multipotent progenitors	0.0493	0.0671	0.2571	0.1518	0.2134	0.1623	0.1902
Erythrocytes	0.0232	0.0324	0.0059	0.0115	0.0065	0.0144	0.8367
Monocytes	0.0152	0.0280	0.0419	0.0451	0.0413	0.0461	0.9440
Macrophages	0.1042	0.0811	0.1951	0.0662	0.1834	0.0657	0.3995
Macrophages M1	0.0209	0.0265	0.0560	0.0318	0.0521	0.0307	0.5545
Macrophages M2	0.0663	0.0718	0.1569	0.0748	0.1536	0.0850	0.8470
Dendritic cells	0.0785	0.0649	0.1049	0.0503	0.0914	0.0555	0.2304
Conventional DC	0.1671	0.0439	0.1149	0.0897	0.1037	0.0856	0.5476
Plasmacytoid DC	0.0515	0.0607	0.1870	0.0773	0.1708	0.0596	0.2733
Immature DC	0.0578	0.0428	0.0482	0.0379	0.0486	0.0370	0.9668
Activated DC	0.0911	0.0651	0.1784	0.0335	0.1662	0.0351	0.0953
Neutrophils	0.0206	0.0368	0.0331	0.0351	0.0320	0.0312	0.8700
Eosinophils	0.1088	0.0855	0.1532	0.1088	0.1791	0.1457	0.3388
Mast cells	0.0239	0.0143	0.0186	0.0129	0.0176	0.0142	0.7460
Basophils	0.1188	0.0938	0.1022	0.1306	0.1264	0.1306	0.3830
Mesenchymal stem cells	0.3516	0.2156	0.2534	0.1972	0.2928	0.2080	0.3579
Adipocytes	0.1497	0.0980	0.1429	0.0747	0.1379	0.0796	0.7611
Preadipocytes	0.0503	0.0423	0.0483	0.0434	0.0548	0.0504	0.5138
Fibroblasts	0.0938	0.0777	0.0547	0.0618	0.0844	0.0910	0.0714
Endothelial cells	0.0899	0.0680	0.0436	0.0484	0.0539	0.0515	0.3319
**Microvascular endothelial cells**	0.0972	0.0639	0.0550	0.0569	0.0824	0.0520	**0.0194**
Lymphatic endothelial cells	0.0980	0.0610	0.0682	0.0579	0.0787	0.0585	0.3928
Smooth muscle cells	0.0707	0.0590	0.0204	0.0324	0.0146	0.0257	0.3574
Skeletal muscle cells	0.0410	0.0169	0.0297	0.0220	0.0279	0.0216	0.6972

Epithelial cells	0.2040	0.0574	0.1642	0.0564	0.1790	0.0499	0.1937
Sebocytes	0.0140	0.0019	0.0160	0.0053	0.0170	0.0047	0.3428
Keratinocytes	0.1121	0.0300	0.1014	0.0389	0.1123	0.0374	0.1809
Mesangial cells	0.0140	0.0179	0.0406	0.0237	0.0445	0.0301	0.4991
Melanocytes	0.0531	0.0367	0.0532	0.0314	0.0628	0.0308	0.1479
Immune Score	0.3821	0.2201	0.6886	0.1766	0.6120	0.1480	**0.0289**
Stroma Score	0.1667	0.0982	0.1206	0.0620	0.1381	0.0804	0.2481
Microenvironment Score	0.5488	0.2030	0.8092	0.1595	0.7500	0.1500	0.0741

VALIDATION COHORT

**Cell type**	**Mean CTLS**	**STDEV CTLS**	**Mean DLE**	**STDEV DLE**	**Mean SCLE**	**STDEV SCLE**	**Mean ACLE**	**STDEV ACLE**	**p-value (DLE v SCLE)**	**p-value (DLE v ACLE)**	**p-value (SCLE v ACLE)**
**B-cells**	0.1360	0.1236	0.4753	0.1424	0.2360	0.1161	0.2849	0.2205	**0.0000**	**0.0025**	0.3864
**naive B-cells**	0.0097	0.0194	0.1487	0.0756	0.0365	0.0514	0.0703	0.0970	**0.0000**	**0.0070**	0.1762
**Memory B-cells**	0.0616	0.0705	0.1490	0.0714	0.0577	0.0571	0.0667	0.0689	**0.0001**	**0.0007**	0.6539
**Class-switched memory B-cells**	0.2708	0.1381	0.4386	0.1170	0.2921	0.1369	0.2948	0.1997	**0.0008**	**0.0084**	0.9609
pro B-cells	0.0061	0.0122	0.0308	0.0510	0.0310	0.0512	0.0269	0.0596	0.9904	0.8254	0.8171
Plasma cells	0.0582	0.0454	0.0492	0.0688	0.0384	0.0432	0.0539	0.0790	0.5542	0.8416	0.4444
CD4+ memory T-cells	0.1568	0.1223	0.5222	0.2897	0.3952	0.2311	0.4969	0.3601	0.1335	0.8072	0.2947
CD4+ naive T-cells	0.1647	0.1914	0.2645	0.1488	0.2203	0.1693	0.2365	0.2310	0.3862	0.6516	0.8014
CD4+ T-cells	0.0398	0.0604	0.2782	0.1981	0.2166	0.1582	0.2922	0.2521	0.2840	0.8466	0.2633
CD4+ Tcm	0.2080	0.1454	0.2283	0.1502	0.1800	0.1250	0.2435	0.1738	0.2759	0.7687	0.1925
CD4+ Tem	0.0785	0.0658	0.2935	0.1787	0.1653	0.1717	0.2921	0.2363	**0.0262**	0.9837	0.0595
CD8+ T-cells	0.1049	0.0595	0.1693	0.1161	0.1186	0.1219	0.1245	0.1199	0.1859	0.2370	0.8787
CD8+ naive T-cells	0.2693	0.0892	0.2620	0.1725	0.2671	0.1727	0.2399	0.1557	0.9258	0.6730	0.6037
CD8+ Tcm	0.0218	0.0366	0.2713	0.1719	0.1486	0.1424	0.1811	0.1595	**0.0187**	0.0936	0.5013
CD8+ Tem	0.0000	0.0000	0.1911	0.1272	0.0873	0.1114	0.1502	0.1476	**0.0092**	0.3541	0.1365
Tregs	0.0201	0.0401	0.1486	0.1189	0.1307	0.1395	0.1596	0.1567	0.6642	0.8049	0.5420
Th1 cells	0.0333	0.0396	0.3016	0.1453	0.2219	0.1549	0.1830	0.1632	0.1017	**0.0201**	0.4448
Th2 cells	0.0946	0.0689	0.1684	0.0959	0.1613	0.0851	0.1421	0.1031	0.8061	0.4084	0.5236
Tgd cells	0.0514	0.0622	0.2269	0.1018	0.1768	0.1020	0.2197	0.1366	0.1284	0.8514	0.2676
NK cells	0.0482	0.0964	0.3396	0.1633	0.2926	0.1569	0.2377	0.1999	0.3593	0.0857	0.3405
NKT	0.7026	0.2604	0.8509	0.4574	0.8194	0.2687	0.8275	0.2750	0.7919	0.8458	0.9250
Hematopoietic stem cells	0.5806	0.2432	0.1624	0.1909	0.2384	0.2458	0.2895	0.2932	0.2814	0.1124	0.5539
Common lymphoid progenitor	0.2938	0.1620	0.3234	0.1315	0.3087	0.1268	0.3384	0.1333	0.7207	0.7225	0.4748
Common myeloid progenitor	0.2625	0.0762	0.0355	0.0573	0.0765	0.1149	0.1075	0.1060	0.1618	**0.0110**	0.3796
Granulocyte-macrophage progenitor	0.0327	0.0653	0.0307	0.0565	0.0907	0.0972	0.0522	0.0767	**0.0220**	0.3198	0.1722
Megakaryocyte-erythroid progenitor	0.0523	0.0923	0.0037	0.0166	0.0051	0.0168	0.0186	0.0605	0.7968	0.2958	0.3424
Multipotent progenitors	0.6266	0.2646	1.1954	0.2888	1.1281	0.3725	0.9742	0.5071	0.5271	0.0983	0.2809
Erythrocytes	0.0000	0.0000	0.0003	0.0015	0.0002	0.0010	0.0000	0.0000	0.7719	0.3236	0.3236
Monocytes	0.0414	0.0828	0.1741	0.1441	0.1655	0.1318	0.1879	0.1617	0.8446	0.7764	0.6328
Macrophages	0.0221	0.0256	0.1899	0.1078	0.1810	0.1046	0.1840	0.1004	0.7929	0.8577	0.9284

Macrophages M1	0.0000	0.0000	0.0968	0.0511	0.0502	0.0570	0.0736	0.0441	**0.0097**	0.1329	0.1542
Macrophages M2	0.0269	0.0538	0.1410	0.1262	0.1858	0.1280	0.1616	0.1465	0.2716	0.6367	0.5803
Dendritic cells	0.1500	0.1122	0.1664	0.1121	0.1679	0.1304	0.2175	0.1688	0.9704	0.2665	0.3043
Conventional DC	0.3299	0.1764	0.1374	0.1586	0.1591	0.1582	0.2518	0.1945	0.6674	**0.0485**	0.1065
Plasmacytoid DC	0.1072	0.1377	0.3653	0.1911	0.2441	0.1394	0.3270	0.1638	0.0275	0.5000	0.0928
Immature DC	0.1360	0.0545	0.0827	0.0851	0.1336	0.1029	0.1243	0.1207	0.0966	0.2155	0.7949
Activated DC	0.0894	0.0654	0.3117	0.0720	0.2619	0.1116	0.3033	0.1126	0.1021	0.7824	0.2498
Neutrophils	0.0474	0.0550	0.0595	0.0500	0.0674	0.0771	0.0859	0.0592	0.7008	0.1354	0.4005
Eosinophils	0.2235	0.1261	0.3219	0.2026	0.3217	0.2443	0.2524	0.1855	0.9970	0.2645	0.3187
Mast cells	0.1025	0.0405	0.0635	0.0379	0.0644	0.0365	0.0717	0.0514	0.9401	0.5730	0.6112
Basophils	0.0168	0.0335	0.1172	0.1376	0.2509	0.2267	0.2260	0.2212	0.0300	0.0697	0.7267
Mesenchymal stem cells	0.4052	0.3514	0.3699	0.3273	0.2997	0.2396	0.3793	0.2889	0.4435	0.9237	0.3487
Adipocytes	0.3322	0.1387	0.2149	0.1454	0.2537	0.1210	0.2443	0.1571	0.3652	0.5425	0.8341
Preadipocytes	0.2823	0.1212	0.2242	0.1108	0.2814	0.1283	0.2157	0.1276	0.1398	0.8226	0.1128
Fibroblasts	0.4991	0.1417	0.1996	0.1895	0.4253	0.2459	0.3942	0.3277	0.0024	0.0271	0.7361
Endothelial cells	0.3063	0.0891	0.0894	0.1054	0.1133	0.1001	0.1213	0.1523	0.4661	0.4463	0.8465
Microvascular endothelial cells	0.3174	0.1455	0.1708	0.1299	0.1874	0.1112	0.1785	0.1561	0.6679	0.8673	0.8368
Lymphatic endothelial cells	0.2609	0.0348	0.1347	0.1025	0.1506	0.0921	0.1289	0.1125	0.6107	0.8644	0.5093
Smooth muscle cells	0.2847	0.0820	0.0115	0.0346	0.0848	0.0690	0.0194	0.0464	**0.0001**	0.5417	**0.0011**
Skeletal muscle cells	0.1043	0.0256	0.0431	0.0418	0.0699	0.0422	0.0669	0.0664	0.0509	0.1830	0.8652
Epithelial cells	0.4073	0.0715	0.3968	0.1701	0.4005	0.1364	0.3588	0.1986	0.9407	0.5193	0.4439
Sebocytes	0.0284	0.0056	0.0428	0.0182	0.0400	0.0141	0.0388	0.0182	0.5877	0.4862	0.8116
Keratinocytes	0.3359	0.0484	0.3332	0.1480	0.3059	0.1158	0.2887	0.1593	0.5186	0.3654	0.6990
Mesangial cells	0.0764	0.0673	0.0264	0.0369	0.0522	0.0579	0.0590	0.0629	0.1012	0.0525	0.7230
Melanocytes	0.0797	0.0514	0.0755	0.0679	0.1227	0.0833	0.1031	0.0760	0.0565	0.2329	0.4407
Immune Score	0.6106	0.3305	1.4919	0.4219	1.2211	0.2812	1.2924	0.5580	**0.0220**	0.2100	0.6130
Stroma Score	0.5688	0.1091	0.2519	0.1736	0.3961	0.1750	0.3799	0.2813	**0.0127**	0.0916	0.8276
Microenvironment Score	1.1794	0.2760	1.7438	0.3997	1.6173	0.3135	1.6723	0.4563	0.2724	0.6011	0.6595
